# COVID-19 pandemic: stringent measures of Malaysia and implications for other countries

**DOI:** 10.1136/postgradmedj-2020-138079

**Published:** 2020-06-19

**Authors:** Sonia Umair, Umair Waqas, Muhammad Faheem

**Affiliations:** School of Business and Economics, Universiti Putra Malaysia, Serdang, Selangor, Malaysia; School of Business and Economics, Universiti Putra Malaysia, Serdang, Selangor, Malaysia; School of Business and Economics, Universiti Putra Malaysia, Serdang, Selangor, Malaysia

In December 2019, pneumonia of unknown cause jolted Wuhan city of Hubei province in China, spread across Asia and the world like wildfire, and, by the end of January 2020, was declared as a public health emergency of international concern by the WHO.^1^ New coronavirus (severe acute respiratory syndrome coronavirus 2 or SARS-CoV-2) was identified as the cause of this disease and was named COVID-19 by the WHO.^1^ The symptoms of COVID-19 include fever, cough and breathing difficulties and can lead to death. These symptoms appear similar to common influenza, but the spread is way far speedier. Despite its low fatality as compared with severe acute respiratory syndrome, its high infection nature has led to a contagion of fear worldwide. Originated in China, the COVID-19 has now been spread across many other countries and has escalated as a global pandemic.^2^ As of 9 April 2020, the COVID-19 outbreaks reached 203 countries, affecting 1 476 819 persons, with 87 816 deaths (84 477 of which are outside China).

In December 2019, pneumonia of unknown cause jolted Wuhan city of Hubei province in China, spread across Asia and the world like wildfire, and, by the end of January 2020, was declared as a public health emergency of international concern by the WHO.^1^ New coronavirus (severe acute respiratory syndrome coronavirus 2 or SARS-CoV-2) was identified as the cause of this disease and was named COVID-19 by the WHO.^1^ The symptoms of COVID-19 include fever, cough and breathing difficulties and can lead to death. These symptoms appear similar to common influenza, but the spread is way far speedier. Despite its low fatality as compared with severe acute respiratory syndrome, its high infection nature has led to a contagion of fear worldwide. Originated in China, the COVID-19 has now been spread across many other countries and has escalated as a global pandemic.^2^ As of 9 April 2020, the COVID-19 outbreaks reached 203 countries, affecting 1 476 819 persons, with 87 816 deaths (84 477 of which are outside China).

China has been the largest trading partner of Malaysia for the last 10 years. In Malaysia, the first COVID-19 case was detected on 25 January in travellers from China arriving via Singapore.^3^ The reported cases in Malaysia remained relatively low until the first wave of cases in late February. After the religious gathering held in Kuala Lumpur in late February and early March, localised clusters began to emerge in Malaysia, and within weeks, Malaysia recorded the largest cumulative number of confirmed COVID-19 cases in Southeast Asia, and by 13 April 2020, the total number of confirmed infections in the country raised to 4817 with a death toll of 77 cases.^4^

Importantly, since the emergence of COVID-19, the Malaysian government has taken serious comprehensive and nationwide measures set by the WHO. Experts across the country are dealing to prevent the spread of the virus widely. For this reason, the Malaysian government has achieved positive results. Here are some major steps, taken by the Malaysian government in response to the COVID-19 epidemic.

The most immediate and direct effect of COVID-19 is on health and healthcare, and it is bound to have outsized economic ramifications. Malaysia started its plan to get prepared for the pandemic well ahead of time. Before the first case, on 20 January 2020, the Health Minister of Malaysia announced that the ministry was now in the process of collecting data on previously ‘non-notifiable’ influenza cases following the emergence of the Wuhan virus.^5^ Initially, the health ministry adopted public health measures of containment. The hospitals were identified to handle the patients, a rapid reverse transcriptase-PCR test on patients and contacts was developed, used and distributed to several government hospitals and medical laboratories, and management protocols were developed.^6^

The second wave of COVID-19 cases spiked on 27 February 2020, and later in mid-March 2020, after an increase in the confirmed cases, the government had to enforce mitigation measures. Initially, the government imposed a 2-week movement control order (MCO) starting from 18 to 31 March, which was extended to 14 April and then to 28 April. The main purpose of this MCO was to flatten the curve of new cases. The prohibition of movement and mass assembly included religious, business, education, sports, culture and social activities except for supermarkets, public markets, grocery stores and stores selling basic necessities.^7^ For community-based control measure, outdoor restriction measures were also enforced, whereby only one resident from a family was allowed to go out at one time and within 10 km of the residence. Checkpoints were set up to check the temperatures at the entry point of residences, supermarkets and food stores. For better prevention, citizens of Malaysia had been prohibited from leaving the country, and foreigners were also not allowed to enter the country.

Due to the slowed-down business activities, the interrupted supply chain of enterprises and personnel quarantine, the outbreak of COVID-19 not only caused health concerns but also had severe impact on the Malaysian economy. Thus, to stable the economic growth, to promote investments and to encourage businesses, the Malaysian government announced an emergency stimulus package, which is among the largest in the world.^8^ When compared with the nation’s gross domestic product, it is about 17%, more than the UK’s 16% and the USA’s 11%.

The public, from entrepreneurs to farmers, from fishermen to those paid daily wages, are concerned about their finances. Thus, the government allocated a special package of RM 10 billion for small and medium-sized enterprises to ease the burden of small and medium enterprises.^9^ The main purpose of this package was to guarantee two-third of the country’s workforce remains employed. The package could give benefits to about 4.8 million workers by giving them a subsidy of Rm 600–Rm 1200.

A 6-month reprieve would be given to individuals and small and medium enterprises, on the repayment of their existing loans. Credit cardholders can choose to convert outstanding balances into term-loans. The corporate sector can restructure their bank loans. This will help to facilitate companies to retain the employment and carry out business activities soonest.

Around the globe, this pandemic caused severe impacts on the education systems. To reduce the delays in education progress, the educational institutes were encouraged to start home-based learning using online classes and other innovative teaching practices. In the future, this can help to develop online educational platforms.^2^ Many universities also showed significant initiatives in taking on social responsibilities by providing food and other necessities to local and international students. International schools issued a subsidised fee for the new term.

According to the early stage of the epidemic, the outbreak started in China and gradually increased its web and captured more than 150 countries by March 2020.^2^ Initially, many of the countries did not take serious actions to control the outbreak in their own countries by thinking it a ‘Chinese virus’. Now governments and organisations are scrambling to manage the disaster caused by COVID-19. Europe and America are paying a high price for their initial ignorance, where America is facing the worst ever medical disaster in their history.^10^

The war against COVID-19 is far from over; however, since the first case, the Malaysian government’s response to the outbreak was epic. On one side, the effective emergency plan launched by the government after just 500 cases across the country has helped to show a significant decline in new cases and a high ratio of recoveries. On the other side, Malaysians, regardless of race, religion and ideology, are trying to help the government and the country is going forward. For this reason, on the 34th day of the MCO, on 20 April 2020, the country reported no COVID-19 deaths, the first time in the month, and the country reported the lowest daily increase of the new cases.^11^

Malaysia’s rapid response to this world pandemic also sets an example for countries that have insecure borders, significant mobile and vulnerable populations, and larger households in denser living conditions. In a study done by a Singapore research agency, Malaysia is ranked fourth in the world out of 105 countries in terms of people’s satisfaction with the government's efforts in dealing with the COVID-19 outbreak.^12^ By 10 May 2020, from 1178 zones around the country, only 0.34% are in red zones, which is a signal of Malaysian victory against COVID-19.

Other countries might need a timely emergency plan to be launched like Malaysia. They can focus on timely lockdown, high level of testing during the lockdown, and try to find clusters among confirmed cases and suspected areas, and cases should be monitored strictly. The government should timely educate the people about the importance of lockdown and the measures needed to be done during this time.^2^ For less infected countries, temperature checking and symptoms among people need to be monitored closely and regularly. Everyone needs to comply with the guidelines, including social distancing and avoiding public gatherings.

‘Resolve, resilience, restart, recovery, revitalise and reform’ is the new normal for the Malaysian people.^13^ It is a six-step plan by the Malaysian government to address the impact of COVID-19 and to make sure that the country emerges stronger despite this virus. Using this six-step plan, Malaysians try to adhere to new standards of operating procedures (SOPs) to prevent another wave of infections in the country and to revive the economy in stages. Despite the loss of millions of dollars, they still have imposed conditional MCO, whereby economic sectors have been allowed to open with enforced guidelines; however, interstate travels are still banned. Red zone areas are strictly being monitored. The education sector has already been shifted to home-based learning in the country. Cinemas, sports competitions, leisure clubs, weddings, social events and theme parks will remain closed. There are special SOPs for houses of all worship faiths. Any changes to the SOPs would be announced from time to time by the government.

In spite of this world pandemic, its approach to international engagement also remains exemplary. To sustain its economy, Malaysia is working closely with China, South Korea, the United Arab Emirates and its ASEAN neighbours to maintain the supply of essential goods and services. Due to fragile borders, collaboration with ASEAN neighbours is vital to sustain virus suppression,^14^ and in a ‘Special ASEAN Summit on COVID-19’, Malaysia proposed a post-COVID-19 economic recovery plan to focus not only on the financial aspects but also on social safety nets, food security and education for the region’s 600 million people. Like every other country tackling the crisis, Malaysia has been fighting hard to survive this pandemic and to rebuild the nation’s economy. Nevertheless, Malaysia’s health response deserves credit for limiting the spread of the virus and minimising avoidable deaths.
